# Virginia

**Published:** 1896-06

**Authors:** 


*Virginia.* Very active discussion, having its chief centre at Richmond, is now going on over tuberculosis among cattle, and the need of milk- and meat-inspection is being thoroughly agitated. The work of examining herds under the recent law passed to control contagious and infectious diseases among live-stock is extending, and its good influences are being felt by a number of communities. One very valuable herd of twenty cows near Richmond, supposed to be in perfect health, revealed on tuberculin-test six diseased ones, all of which were found affected when destroyed.

A very interesting question has arisen in this State in a herd of very valuable cattle, tested some two years ago by representatives of the Bureau of Animal Industry, when some responded, but were not all destroyed. Recently, under the pressure of public opinion, this same herd was again tested, and a number of those which responded at the first test are said to have failed on the second test, and the question naturally arises, How many of these should be taken from the herd and destroyed? If the first test was a reliable and trustworthy one, the question naturally arises, Have these animals recovered from that one injection or was the last test an imperfect one?

